# Bovine Tuberculosis in Buffaloes, Southern Africa

**DOI:** 10.3201/eid1605.090710

**Published:** 2010-05

**Authors:** Michel de Garine-Wichatitsky, Alexandre Caron, Calvin Gomo, Chris Foggin, Keith Dutlow, Davies Pfukenyi, Emily Lane, Sébastien Le Bel, Markus Hofmeyr, Tiny Hlokwe, Anita Michel

**Affiliations:** Centre de Coopération Internationale en Recherche Agronomique pour le Développement, Harare, Zimbabwe (M. de Garine-Wichatitsky, A. Caron, S. Le Bel); Centre de Coopération Internationale en Recherche Agronomique pour le Développement, Montpellier, France (M. de Garine-Wichatitsky, S. Le Bel); University of Pretoria, Pretoria, South Africa (A. Caron, A. Michel); University of Zimbabwe, Harare (C. Gomo, D. Pfukenyi); Department of Veterinary Services, Harare (C. Foggin, K. Dutlow); National Zoological Gardens of South Africa, Pretoria (E. Lane); South Africa National Parks, Kruger National Park, South Africa (M. Hofmeyr); Agricultural Research Council–Onderstepoort Veterinary Institute, Pretoria (T. Hlokwe, A. Michel)

**Keywords:** Bovine tuberculosis, tuberculosis and other mycobacteria, wild animals, buffaloes, transboundary spread, southern Africa, letter

**To the Editor**: Emergence of bovine tuberculosis (TB) in wildlife in southern Africa has implications not only for the conservation of the wildlife species affected (*1*) but also for the health of humans and livestock living at the wildlife–livestock–human interface (*2*). Bovine TB in South Africa’s Kruger National Park was first found in African buffaloes (*Syncerus caffer*) in 1990 (*3*) and likely entered the park by cattle-to-buffalo transmission (*4*). Bovine TB infection has been spreading northward; in 2003, infection was confirmed in a buffalo ≈60 km south of the Limpopo River. In 2005, a case was confirmed only 6 km south of the river (D. Keet, unpub. data). In 2008, we isolated *Mycobacterium bovis* from African buffaloes in Zimbabwe.

During October 9–13, 2008, a total of 38 buffaloes from 4 herds were captured in Gonarezhou National Park (south of the Mabalauta area; 22.0553°S, 31.4265°E). Blood samples were collected, sampled buffaloes were marked and released, and 3 adult females in each herd were equipped with radio collars. Buffalo tissue samples were collected, packaged, shipped, and handled at the Agricultural Research Council–Onderstepoort Veterinary Institute according to procedures recommended for controlling the spread of foot-and-mouth disease virus. Interferon-γ assay (*5*) results were positive for bovine TB for 4 (10.5%) buffaloes: 2 adult females and 1 young adult male from the same herd and 1 adult female from another herd.

Four months later, a radio-collared adult female and the young adult male, each of which had had positive interferon-γ assay results, were sedated and euthanized, and necropsies were performed in the field. Samples for histopathologic examination and culture were collected from lymph nodes of the head and thorax. No acid-fast organisms were detected, but the histologic findings were strongly suggestive of paucibacillary TB. *M. bovis* was isolated from the retropharyngeal lymph nodes of both buffaloes and from the bronchial and head lymph nodes of 1 of them. Both isolates were typed by analysis of variable number of tandem repeat (VNTR) sequences at 6 loci (exact tandem repeat A–F) (*6*) and compared with the VNTR profiles of ≈75 isolates from Kruger. All isolates showed an identical VNTR profile (7544*52.3), which suggests an epidemiologic link between the *M. bovis* infections in the 2 parks. However, the exact tandem repeat loci had lower discriminatory power among Kruger isolates than did IS*6110* restriction fragment length polymorphism typing (T. Hlokwe, unpub. data) (*4*). A typing regimen comprising different typing methods and markers will be useful for more accurately determining the genetic relationship between the isolates from the 2 parks, Gonarezhou and Kruger.

The confirmation of results for bovine TB–infected buffaloes in Zimbabwe (Gonarezhou National Park) raises several questions regarding the transboundary spread of animal disease and has considerable management implications for the Great Limpopo Transfrontier Conservation Area. The most likely scenario is buffalo-to-buffalo contact across the boundary because the bovine TB cases reported here were located <45 km from the unfenced northern boundary of Kruger National Park. Buffaloes, especially bulls and young heifers, frequently move from herd to herd and may contribute to the spread of *M. bovis* by mixing with unexposed herds (*7*). Although transboundary movements of buffaloes between Kruger and Gonarezhou have not been specifically documented, uncontrolled movements across the Limpopo River do occur (de Garine-Wichatitsky, unpub. data). However, >12 wild species in Kruger have now been found to be infected by bovine TB (*2*). Most of these species are probably not effective sources of *M. bovis* infection for buffaloes, but the disease epidemiology could rely on multihost reservoirs (*8*). Thus, a second scenario could be a buffalo-to–unidentified wild species–to-buffalo pathway, because species like greater kudu (*Tragelaphus strepsiceros*) appear to be able to maintain, spread, and even drive a bovine TB epidemic (*4*,*9*). A third scenario involves movement of infected livestock across the boundaries of the 3 countries of the Great Limpopo Transfrontier Conservation Area, resulting in cattle-to-buffalo transmission of bovine TB. As a last scenario, we cannot rule out the possibility that bovine TB infection of buffaloes has remained silent and undetected for decades in Zimbabwe.

The management implications of bovine TB in buffaloes in Gonarezhou National Park are considerable. Once bovine TB is established in a native free-ranging maintenance host, eradication is unlikely (*2*,*10*). Evaluation of the prevalence and distribution of the infection in wildlife and livestock populations on the Zimbabwe side of the Great Limpopo Transfrontier Conservation Area is urgently needed. Control options in wildlife are limited (*2*,*10*), but chances of success are greater if control measures are initiated at the early stage of disease spread into a new area. Adequate risk-mitigation strategies should be developed and implemented to reduce the risk for bovine TB transmission to livestock and humans living at the periphery of the unfenced Gonarezhou National Park. Failure to promptly assess the situation and adopt appropriate measures would have far-reaching conservation, economic, and public health consequences, not only for Zimbabwe but also for the political and social acceptance of the transfrontier conservation areas in southern Africa.
